# Editorial: Critical issues and hot topics in endovascular repair of aortic dissection

**DOI:** 10.3389/fcvm.2024.1396852

**Published:** 2024-03-21

**Authors:** Jiaxue Bi, Xiangchen Dai

**Affiliations:** ^1^ Department of Vascular Surgery, Tianjin Medical University General Hospital, Tianjin, China; ^2^ Tianjin Key Laboratory of Precise Vascular Reconstruction and Organ Function Repair, Tianjin, China

**Keywords:** aortic dissection, endovascular repair, complication, treatment, editorial

**Editorial on the Research Topic** Critical issues and hot topics in endovascular repair of aortic dissection

Over the last decades, great advancement has been achieved in endovascular repair of aortic dissection. Endovascular repair for type B aortic dissections has been widely accepted and proven as a safe and effective treatment. With the continuous innovation of surgical instruments and methods, endovascular repair has also achieved promising results in the treatment of type A aortic dissection, although open repair is still the gold standard. However, there are specific problems associated with endovascular repair, including retrograde type A aortic dissection, distal stent-graft-induced new-entry, various types of endoleaks, stent-graft migration and collapse, and dilation of the distal residual false lumen. These problems are closely related with prognosis. As endovascular repair becomes the mainstream and long-term complications in early patients begin to become prominent, how to reasonably choose surgery, properly manage long-term complications, and improve the prevention and management of aortic dissection are the directions that need more attention and investigation in the future. The articles featured in this collection attempt to address some of these important topics and provide readers with new insights into the current diagnosis and treatment of aortic dissection.

Zhao et al. explored the optimal timing of endovascular repair of uncomplicated type B aortic dissection (uTBAD) in a meta-analysis of 6 studies involving 3,769 patients, suggesting that the early prognosis of endovascular repair in the subacute phase appears to be superior to that in the acute phase, although there is no significant difference in long-term prognosis in the timing of surgery. Although thoracic endovascular aortic repair (TEVAR) is still recommended in the subacute or chronic phase of uTBAD according to current guidelines, and early intervention with TEVAR should only be considered in selected cases (1–3). However, it is well known that aortic disease is a highly dynamically developing pathology, with aneurysm degeneration and rupture that can progress suddenly within a few days to months with catastrophic consequences. For the temporarily stable uTBAD, the latent and unknown dangers test the surgeon's wisdom in deciding the timing of surgery. Erin et al. (4) demonstrated by meta-analysis the safety of TEVAR in the acute phase from 2 to 14 days after the onset of uTBAD. They believe that there is a probably a subgroup of patients with uTBAD with at risk features that would most benefit from early intervention with TEVAR between 2 and 14 days of symptom onset without increased mortality or morbidity risk. Early dissection flaps are both soft and fragile, so the choice of the best timing for surgery is partly a trade-off between aortic fragility and aortic remodeling. The potential timing of early TEVAR should be carefully considered based on clinical, anatomical, and patient factors to provide a platform for improved aortic remodelling in the long term without added mortality risk.

Reconstruction of supra-arch branches is a difficult and hot topic in endovascular repair of the aorta, and the branched TEVAR is arguably one of the most promising. The Castor stent graft is a commercially available single-branch stent that has demonstrated excellent performance and good results in the reconstruction of left subclavian artery (LSA) during aortic dissection therapy. The results of the meta-analysis by Yao et al. showed that, compared with chimney technology, fenestration technology, and hybrid technology, Castor stent graft technology has the advantages of higher technical success rate and long-term patency rate of the LSA, as well as lower mortality rate and endoleak rate. Several single-branch stentgrafts of different designs are available (5–7), but slow research progress and unsatisfactory clinical trial results signal that this is still a long and challenging road. At the same time, the development of multibranch stentgrafts is also in full swing, and good early trial results show us the possibility of total endovascular technology to resolve thoracic aortic diseases, such as aortic dissection, and the arrival of a new era of minimally invasive aortic treatment (8, 9).

An et al. identified risk factors for in-hospital mortality after total arch procedure in patients with acute type A aortic dissection in a single-center retrospective study. Yang et al. screened for risk factors for in-hospital death from TBAD in a retrospective study and developed a predictive model enabling clinicians to provide individualized patient management. The development of disease risk factors and predictive models is valuable in guiding clinical practice. Although the study contained a large cohort of patients, the lack of externally validated single-center data could lead to bias and insufficient generalizability of the results. There are many similar predictive models based on single-center data with good accuracy, but it is difficult for other centers to trust these models in clinical practice other than their own. There is an urgent need to bring together multi-center and large patient cohorts, and integrate the appropriate advanced machine learning algorithms and even artificial intelligence to build trustworthy and pervasive predictive models.

In addition, Zhao et al. shared the clinical features and treatment strategies of rare spontaneous isolated abdominal aortic dissection (SIAAD), indicating that the clinical features of SIAAD vary depending on the location of the primary entry tear and the number of dissected aortic zones, and although surgical treatment was not associated with higher survival rate of SIAAD, it is associated with a lower incidence of false lumen progression and a higher rate of aortic remodeling. The clinical presentation of IAAD is highly variable. It ranges from incidental discovery without symptoms to serious complications with visceral or limb ischemia, which may cause decision-making difficulties for standard initial treatment strategies (10). Therapeutic strategies are conservative in the asymptomatic form with a non-dilated aorta, while open or endovascular repair are the treatment of choice in the symptomatic cases. This decision is greatly influenced by anatomical conditions along with the surgeon's experience. There is an urgent need for a normative consensus to guide clinical practice.

Endovascular aortic repair has played and continues to play an increasing role in the treatment of patients with aortic dissection. Despite the abundance of reports and literature on the topic, there is still a great deal of controversy among experts in the field regarding the timing and technique of surgery and the assessment of prognostic risk factors, among other things. Treatment options are certainly evolving and will be heavily influenced by patient-related factors, anatomy, and lesion characteristics. We now have many techniques and concepts for treating aortic dissection and other lesions, and the next step is to realize how to utilize them. The heterogeneity of individual patients also means the heterogeneity of patients' lesions, and each patient should have their own treatment plan, and personalized disease management will be the next step in our efforts. Hopefully, these articles will help clarify some of the existing controversies.

## References

[1] Isselbacher EM Preventza O Hamilton Black J 3rd Augoustides JG Beck AW Bolen MA 2022 ACC/AHA guideline for the diagnosis and management of aortic disease: a report of the American Heart Association/American College of Cardiology joint committee on clinical practice guidelines. Circulation. (2022) 146(24):e334–482. 10.1161/CIR.0000000000001106 36322642 PMC9876736

[2] Hameed I Cifu A S Vallabhajosyula P. Management of thoracic aortic dissection. JAMA. (2023) 329(9):756–7. 10.1001/jama.2023.0265 36795378

[3] Riambau V Böckler D Brunkwall J Cao P Chiesa R Coppi G Editor’s choice—management of descending thoracic aorta diseases: clinical practice guidelines of the European Society for Vascular Surgery (ESVS). Eur J Vasc Endovasc Surg. (2017) 53(1):4–52. 10.1016/j.ejvs.2016.06.005 28081802

[4] Saricilar EC Patel K Gatmaitan R Puttaswamy V. Editor’s choice—optimal timing of thoracic endovascular aortic repair for uncomplicated type B aortic dissection: a systematic review and meta-analysis. Eur J Vasc Endovasc Surg. (2023) 65(6):851–60. 10.1016/j.ejvs.2023.02.080 36871923

[5] Rousseau H Revel-Mouroz P Saint Lebes B Bossavy J P Meyrignac O Mokrane FZ. Single aortic branch device: the Mona LSA experience. J Cardiovasc Surg (Torino). (2019) 60(1):81–90. 10.23736/S0021-9509.18.10665-3 30001611

[6] Dake MD Fischbein MP Bavaria JE Desai ND Oderich G Singh MJ Evaluation of the gore TAG thoracic branch endoprosthesis in the treatment of proximal descending thoracic aortic aneurysms. J Vasc Surg. (2021) 74(5):1483–90.2. 10.1016/j.jvs.2021.04.025 33940079

[7] Planer D Elbaz-Greener G Mangialardi N Lindsay T D'Onofrio A Schelzig H NEXUS Arch: a multicenter study evaluating the initial experience with a novel aortic arch stent graft system. Ann Surg. (2023) 277(2):e460–6. 10.1097/SLA.0000000000004843 33714965

[8] Zhang H Rong D Liu F Ge Y Wei R Zhu Y Case series of total endovascular repair of the aortic arch with a modular inner-branched stent-graft system: first-in-man experience. Br J Surg. (2023) 110(9):1084–6. 10.1093/bjs/znad070 37075484 PMC10416682

[9] Tenorio ER Oderich GS Kölbel T Dias NV Sonesson B Karelis A Multicenter global early feasibility study to evaluate total endovascular arch repair using three-vessel inner branch stent-grafts for aneurysms and dissections. J Vasc Surg. (2021) 74(4):1055–65.4. 10.1016/j.jvs.2021.03.029 33865950

[10] Liu Y Han M Zhao J Kang L Ma Y Huang B Systematic review and meta-analysis of current literature on isolated abdominal aortic dissection. Eur J Vasc Endovasc Surg. (2020) 59(4):545–56. 10.1016/j.ejvs.2019.05.013 31822385

